# Could a *Shigella* vaccine impact long-term health outcomes?: Summary report of an expert meeting to inform a *Shigella* vaccine public health value proposition, March 24 and 29, 2021

**DOI:** 10.1016/j.jvacx.2022.100218

**Published:** 2022-09-21

**Authors:** Karoun H. Bagamian, Chloe Puett, John D. Anderson, Farzana Muhib, Clint Pecenka, Jere Behrman, Robert F. Breiman, Ijeoma Edoka, Susan Horton, Gagandeep Kang, Karen L. Kotloff, Claudio F. Lanata, James A. Platts-Mills, Firdausi Qadri, Elizabeth T. Rogawski McQuade, Christopher Sudfeld, Pascale Vonaesch, Thomas F. Wierzba, Suzanne Scheele

**Affiliations:** a Bagamian Scientific Consulting, LLC, Gainesville, FL 32601, USA; b Department of Environmental and Global Health, University of Florida, Gainesville, FL 32603, USA; c Stony Brook University, Department of Family, Population & Preventative Medicine, Program in Public Health, Stony Brook, NY 11794, USA; d Center for Vaccine Innovation and Access, PATH, 455 Massachusetts Ave NW, Washington, DC 20001, USA; e Center for Vaccine Innovation and Access, PATH, Seattle, WA 98121, USA; f Department of Economics and Sociology, University of Pennsylvania, Philadelphia, PA 19104, USA; g Hubert Department of Global Health, Rollins School of Public Health, Emory University, Atlanta, GA 30322, USA; h Health Economics and Epidemiology Research Office, Department of Internal Medicine, School of Clinical Medicine, Faculty of Health Sciences, University of the Witwatersrand, Johannesburg, South Africa; i School of Public Health, Faculty of Health Sciences, University of the Witwatersrand, Johannesburg, South Africa; j School of Public Health Sciences, University of Waterloo, Waterloo, ON N2L 3G1, Canada; k Division of Gastrointestinal Sciences, Christian Medical College, Vellore 632004, India; l Department of Pediatrics, Center for Vaccine Development and Global Health, University of Maryland School of Medicine, Baltimore, MD, USA; m Instituto de Investigacion Nutricional, Lima, Peru; n Division of Infectious Diseases & International Health, University of Virginia, Charlottesville, VA 22903, USA; o International Centre for Diarrhoeal Disease Research, Bangladesh (icddr,b), Dhaka, Bangladesh; p Department of Epidemiology, Emory University, Atlanta, GA 30322, USA; q Department of Global Health and Population, Harvard T.H. Chan School of Public Health, Boston, MA 02115, USA; r Department of Fundamental Microbiology, University of Lausanne, Switzerland; s Department of Internal Medicine, Section on Infectious Diseases, Winston-Salem, NC 27157, USA

**Keywords:** *Shigella*, Childhood diarrhea, Growth faltering, Vaccine, Economic model, Cost-benefit, stunting

## Abstract

Shigellosis is a leading cause of diarrhea and dysentery in young children from low to middle-income countries and adults experiencing traveler’s diarrhea worldwide. In addition to acute illness, infection by *Shigella* bacteria is associated with stunted growth among children, which has been linked to detrimental long-term health, developmental, and economic outcomes. On March 24 and 29, 2021, PATH convened an expert panel to discuss the potential impact of *Shigella* vaccines on these long-term outcomes. Based on current empirical evidence, this discussion focused on whether *Shigella* vaccines could potentially alleviate the long-term burden associated with *Shigella* infections. Also, the experts provided recommendations about how to best model the burden, health and vaccine impact, and economic consequences of *Shigella* infections. This international multidisciplinary panel included 13 scientists, physicians, and economists from multiple relevant specialties.

According to the panel, while the relationship between *Shigella* infections and childhood growth deficits is complex, this relationship likely exists. Vaccine probe studies are the crucial next step to determine whether vaccination could ameliorate *Shigella* infection*-*related long-term impacts. Infants should be vaccinated during their first year of life to maximize their protection from severe acute health outcomes and ideally reduce stunting risk and subsequent negative long-term developmental and health impacts. With vaccine schedule crowding, targeted or combination vaccination approaches would likely increase vaccine uptake in high-burden areas. *Shigella* impact and economic assessment models should include a wider range of linear growth outcomes. Also, these models should produce a spectrum of results—ones addressing immediate benefits for usual health care decision-makers and others that include broader health impacts, providing a more comprehensive picture of vaccination benefits. While many of the underlying mechanisms of this relationship need better characterization, the remaining gaps can be best addressed by collecting data post-vaccine introduction or through large trials.

## Introduction

Infection by *Shigella* enterobacteria is a leading cause of diarrhea and dysentery among children younger than five years of age in low- and middle-income countries (LMICs) and adults experiencing traveler’s diarrhea. Shigellosis is the second-leading cause of diarrheal mortality for people of all ages, estimated to be responsible for approximately 63,000 deaths among children under five each year globally [1]. *Shigella* spp., endemic in temperate and tropical areas, are most often spread from fecal-oral transmission, especially in environments with inadequate access to sanitation and hygiene. *Shigella* spp. are also highly contagious and have a low infectious dose, spreading even in higher socioeconomic status (SES) settings with inadequate hygiene practices. *Shigella*-attributable diarrhea is most common in toddlers (12 to 24 months) and young children (25 to 59 months) [4], [13], [14], [17], [18], [21]. Despite a lower *Shigella* incidence during infancy, infants are more likely to experience more severe illness from *Shigella* infection [21], [22], [23]. In addition to their acute health impacts, *Shigella* infections have been repeatedly identified to have a relationship to stunted linear growth during childhood, especially among children in countries with inadequate access to appropriate water, sanitation, and hygiene (WASH) [2], [3], [4], [5], [6], [7].

Estimates from 2020 indicate that 149.2 million children under five worldwide have stunted growth [8], with millions more experiencing some level of linear growth faltering. Linear growth faltering is when a child’s height falls below the expected growth curve [9]. This term is most often used when assessing linear growth deficits using continuous child’s length or height-for-age Z-score (LAZ; HAZ) outcomes. Stunting is when a child’s LAZ or HAZ is more than two standard deviations below the World Health Organization’s (WHO’s) Child Growth Standards median and is the term for describing linear growth faltering as a dichotomous categorical outcome. Linear growth faltering and childhood stunting are indicators of chronic undernutrition and are associated with increased morbidity and mortality during childhood. They are linked to myriad downstream outcomes, such as diminished physical, motor, and cognitive development during childhood, as well as poor chronic health status and decreased earning potential as adults [10], [11], [12].

Historically, most studies linking *Shigella* and other enteric pathogens to diminished childhood growth estimated this association between symptomatic enteric infections (any-cause diarrheal cases, usually moderate-to-severe diarrheal episodes) and subsequent anthropometry [2], [3], [3], [5], [17]. The increased use of new, more sensitive diagnostic methods in recent studies [4], [13], [14] indicate that *Shigella*’s burden in LMICs is likely far greater than previously thought. These studies have also reported associations between childhood linear growth deficits and less severe any-cause diarrheal episodes [4], [18] and even asymptomatic infections [4], [19], [20] attributed to *Shigella* and other enteric pathogens. The increased detection of *Shigella* in young children, coupled with the bacterium’s ability to cause severe symptoms, its increasing resistance to multiple antibiotics [15], and its role in exacerbating undernutrition and linear growth faltering, indicates that *Shigella* is a prime vaccine target. The current recommended treatment of dysentery further prompts the need for *Shigella* vaccines. WHO guidelines recommend antibiotic treatment of dysentery, and proposed antibiotic stewardship interventions will not reduce their use for this symptom. These circumstances are problematic because although dysentery is classically associated with *Shigella*, it is also a symptom of infection by other pathogens, and not all *Shigella* infections present with dysentery, which can result in over or undertreatment with antibiotics. Preventative measures, including *Shigella* vaccines, are needed to avoid the treatment-prompting symptoms that may result in antibiotic misuse. No *Shigella* vaccines are available yet, but several candidates are in clinical development [16].

Previous studies [39], [40] surveying the cost-effectiveness of a potential *Shigella* vaccine have shown it to be less cost-effective than other enteric vaccines recently introduced by LMIC vaccination programs [41], [42], even when accounting for *Shigella*-attributable stunting impacts [39]. However, accounting for these long-term impacts boosted the vaccine’s cost-effectiveness, especially for specific regions. Since these models were published, additional studies have supported *Shigella*’s potential role in long-term effects on child growth and other aspects of this relationship (e.g., its relationship to gut inflammation markers) [6], [21], [43]. These studies suggest broadening burden envelopes to include the full spectrum of *Shigella*’s long-term effects on child growth and future productivity may be warranted. However, to do this responsibly, modelers must ensure that they are considering essential nuances about stunted growth and future non-health-related impacts. For example, while stunted growth has been linked to poor cognitive and educational outcomes in many settings [31], [32], [33], [34], [35], precisely measuring this relationship is difficult because many genetic and environmental factors are involved. Current evidence supports only an associative, rather than causal, relationship between them [36], [37], [38]. Regardless of these measurement challenges, the relationship between stature and adult health and economic outcomes remains of great research interest.

A crucial step in understanding the value of *Shigella* vaccines is determining the potential public health impact and economic value of vaccination. A part of this effort is exploring whether the *Shigella* vaccine’s hypothesized influence on childhood growth faltering would significantly affect its overall health impact. On March 24 and 29, 2021, PATH convened an expert panel to discuss these issues and provide evidence-based recommendations about outstanding knowledge gaps related to the potential impact of a *Shigella* vaccine on childhood stunting and associated long-term economic consequences. In addition, the panel explored how to best update existing vaccine impact and cost-effectiveness models and prepare for additional economic analyses that incorporate the effects of *Shigella*-attributable stunting throughout the life course. The panel comprised 13 scientists, physicians, and economists from seven countries, who provided expertise ranging from the epidemiology and immunology of enteric diseases to the economic modeling of childhood health outcomes, encompassing vaccine development, nutrition, child development, and other relevant specialties (Table 1).

**Table 1:** Expert panel, relevant expertise, and affiliations.

**Names**	**Expertise**	**Affiliation**
Robert Breiman, MD (Chair)	Pediatric infectious disease, surveillance, and vaccine advisory	Hubert Department of Global Health, Rollins School of Public Health, Emory University, Atlanta, USA

Jere Behrman, PhD	Economics/sociology, emphasizing nutritional early childhood development	Department of Economics and Sociology, University of Pennsylvania, Philadelphia, PA, USA

Ijeoma Edoka, PhD	Econometrics and economic evaluation of healthcare interventions	Health Economics and Epidemiology Research Office, Department of Internal Medicine, School of Internal Medicine, School of Clinical Medicine, Faculty of Health Sciences School of Public Health, Faculty of Health Sciences University of Witwatersrand Johannesburg, South Africa

Susan Horton, PhD	Global health economics, economics of nutrition	School of Public Health Science, University of Waterloo, Waterloo, ON Canada

Gagandeep Kang, MD, PhD, FRS	Vaccines and public health, with a focus on pediatric enteric infections in India	Division of Gastrointestinal Sciences, Christian Medical College, Vellore, India

Karen Kotloff, MD	Infectious disease epidemiology and vaccine development	Department of Pediatrics, Center for Vaccine Development and Global Health, University of Maryland School of Medicine, Baltimore, MD, USA

Claudio Lanata, MD, MPH	Nutrition and public health, infectious disease	Instituto de Investigacion Nutricional Lima, Peru

James Platts-Mills, MD	Molecular diagnostics and epidemiology of pediatric enteric diseases	Division of Infectious Diseases & International Health, University of Virginia, Charlottesville, VA, USA

Firdausi Qadri, PhD	Infectious disease immunology and vaccine development	International Centre for Diarrheal Disease Research, Dhaka, Bangladesh

Elizabeth Rogawski McQuade, PhD, MSPH	Infectious disease epidemiology, focus on pediatric enteric infections and environmental enteropathy	Department of Epidemiology, Emory University, Atlanta, GA, USA

Christopher Sudfeld, ScD	Epidemiologist focusing on interaction of nutrition and infection on maternal and child health	Department of Global Health and Population Harvard University, Harvard T.H. Chan School of Public Health, Boston, MA, USA

Pascale Vonaesch, PhD, MSc, MPH	Microbiologist and infection biologist focused on microbiota and wider gut ecosystem in child nutrition and enteric disease	Department of Fundamental Microbiology, University of Lausanne Swiss Tropical & Public Health Institute, Switzerland

Thomas Wierzba, PhD, MS, MPH	Vaccine development in pediatric enteric disease	Department of Internal Medicine, Section on Infectious Diseases, Wake Forest University Winston-Salem, NC, USA

Many of the questions posed to the experts inspired meaningful conversations about *Shigella* infections, childhood stunting, and the role of WASH and enteric vaccine interventions. The discussion addressed two main overarching themes: the relationship(s) between (1) *Shigella* and childhood growth faltering/stunting and (2) childhood growth faltering/stunting and long-term economic impacts. Panel members were asked several predetermined questions related to each theme. This report summarizes the critical discussions, emergent relevant discussion themes, recommendations, and suggested future research avenues from this expert meeting. In this report, we use the term “any-cause” diarrhea for etiology agnostic results and “*Shigella*-attributable” diarrhea for results specific to *Shigella*.

## Session 1. The relationship between *Shigella* and childhood growth faltering and stunting

***Question 1: Are you confident that there is a relationship between Shigella and childhood linear growth deficits? Do you think that less severe Shigella-attributable diarrhea or asymptomatic infections also contribute to stunting risk? If so, what would be the range or upper bound of the impact of Shigella on a child’s growth?***

The panel members agreed that there was a relationship between experiencing *Shigella*-related disease and diminished child growth. While they thought an association between less severe *Shigella* infections and linear growth faltering exists, they were concerned about the limited evidence regarding asymptomatic detections and growth faltering. Most evidence supporting this relationship is around short-term growth effects approximately 60 to 90 days after a diarrheal episode [4], [7], leaving the association between longer-term growth outcomes and asymptomatic detections less well-established. However, short-term faltering likely has clinical significance. In one large multisite study, children with any-cause moderate-to-severe diarrhea (MSD) experienced 8.5 times higher mortality at an approximately 60-day follow-up than children without MSD [17].

The panel discussed the evidence and limitations concerning the relationship between less severe/subclinical *Shigella* infections and linear growth faltering. To date, two extensive multisite studies in young children from LMICs have linked less severe enteric infections of several etiologies (including *Shigella*) to linear growth deficits [4], [18]. As for asymptomatic/subclinical *Shigella* infections and linear growth deficits, one large-scale multisite study [4] and one single-site study [20] have reported this association, while another single-site study did not find an association [6]. While bacterial culture methods can detect asymptomatic infections, these types of infections are more easily identifiable by more recently available molecular diagnostic approaches. Because subclinical disease cases are, or appear to be, asymptomatic, their systematic detection is difficult unless the experimental design includes active surveillance.

Another concern expressed by panel members was that previous studies of the relationship between *Shigella* and linear growth have used varying metrics and approaches to assign diarrheal etiology and symptomology and assess child linear growth outcomes, making it difficult to standardize the effect of *Shigella* infection across studies. They noted that global data on asymptomatic *Shigella* detection in relation to childhood growth does not currently exist, making it difficult to include such infections in current vaccine benefit and impact models.

The panel discussion did not lead to a definitive answer regarding the likely magnitude (or range) of the associations of asymptomatic/subclinical *Shigella* detections and linear growth faltering because of the inherent difficulties in measuring this relationship and the likely heterogeneity in effect by site and age. However, the experts most directly involved in empirically studying the association between *Shigella* infections and growth faltering suggested that, based on their results comparing growth in children with high vs. low *Shigella* detection burden, a reasonable upper bound might be a 0.15 LAZ score decrement for young children up to 24 months old. Based on Rogawski et al. [4], a plausible estimate of the effect of a single *Shigella* episode would be a 0.03 (range 0.00 to 0.05) LAZ score decrement per *Shigella*-attributable diarrheal episode for children under two years of age.

***Question 2. Is a single Shigella infection insufficient (requiring repeated infections or poor nutrition) to impact linear growth? Would preventing infection—or inflammatory or another process that a single infection may cause—directly reduce linear growth faltering? Might other pathogens at least partially replace the effect on linear growth?***

Considering the body of evidence, the panel identified several important factors to include in future analyses examining the relationship between shigellosis and growth faltering. They supported modeling approaches that account for repeated infections and cumulative burden. Also, they pointed out the importance of accounting for infection duration, as studies have shown *Shigella’s* association with prolonged acute diarrhea (duration 7 to 13 days) and persistent diarrhea (duration ≥ 14 days) [25], [26], and longer episodes exacerbate undernutrition and other adverse illness outcomes [26]. Some panel members suggested that it was essential to assess how antibiotic treatment of *Shigella* episodes may influence child growth and that it was worth exploring its potential influence on model outcomes through sensitivity analyses. They cited recent findings that WHO-recommended antibiotic treatment of *Shigella*-positive MSD ameliorated the relationship between *Shigella* and growth deficits in children under two [7]. Finally, the panel suggested that models should account for the common occurrence of co-infection with multiple enteric pathogens, as there are different ways of assigning primary and secondary etiologies of these episodes.

As discussed by the panel, models assessing the impact of a *Shigella* vaccine on stunting should account for the likely replacement of *Shigella* by other enteric pathogens that can impact child growth. *Shigella* is just one of many enteric pathogens (*Escherichia coli* spp*.*, *Campylobacter* spp*., Cryptosporidium* spp*.*, *Giardia* spp*.*, and *Enterocytozoon bieneusi*) that have been linked to linear growth deficits [4], [17], [18], [27]. A panel member asserted that in a recent multisite longitudinal birth cohort study, the decreases in linear growth associated with certain enteric pathogen detections (including *Shigella*) were not additive—implying that removing one growth faltering-related pathogen while others are present might have only a marginal effect on linear growth.

The panel members expressed doubt about a *Shigella* vaccine being able to prevent subclinical infections. Like most vaccines, a *Shigella* vaccine would most likely avert disease symptoms or severe illness but whether it could provide sterilizing immunity against *Shigella* infection remains unknown. For example, in one *Shigella* vaccine trial, when challenged with a *Shigella* inoculum, vaccinated human volunteers were protected from illness symptoms but not infection [28]. While it is theoretically possible that a *Shigella* vaccine could prevent infection, reduce the asymptomatic infection burden, and result in less stunting, this relationship has not been tested yet.

***Question 3. Is there convincing evidence that preventing or attenuating Shigella infections by vaccinating infants younger than nine months would significantly impact childhood stunting?***

Panel members agreed that early *Shigella* vaccination is needed to provide maximum protection from severe disease in infants and ideally prevent future stunting. *Shigella*-related mortality is highest during the first year of life [22], [29]. Also, the first 24 months of a child’s life are considered the most important in determining a child’s future growth and development, with the steepest diarrheal-attributed decreases in growth occurring during infancy [30]. A panel member stated that a recent diarrheal etiology study in Niger found approximately 60% of severe shigellosis in infants [23], highlighting the importance of early vaccination of children from countries with high endemic *Shigella* burden to maximize their protection from infection. The panel members agreed that while preventing *Shigella* infections is expected to reduce childhood growth deficits or stunting, vaccine probe trials that include measures of growth faltering as an *a priori* outcome are needed.

**Emergent discussion theme: Ideal vaccination schedules and potential crowding solutions**

During the discussion of Question 3, a theme concerning ideal vaccination schedules emerged. This discussion and the solutions presented by panel members are summarized here.

As *Shigella* incidence is high throughout the first five years of life [13], [17], panel members asserted that the ideal *Shigella* vaccination schedule would have to simultaneously provide immunity as early as possible (probably close to six months of age) that ideally lasts for up to five years, protecting children during their most vulnerable period. Accordingly, most members felt that future *Shigella* vaccination schedules should begin by six or seven months. They agreed that vaccinating children at six and nine months would provide immunity after the period of protection likely conferred by passive antibody transfer during breastfeeding [22], [24] and avoid vaccine schedule crowding (see next paragraph). However, the panel considered vaccinating infants at three and six months ideal for maximizing protection, especially in high endemic burden settings. Regional variations in vaccination schedules (e.g., in Africa vs. Latin America) would likely emerge, as the schedule would be deployed according to the risk of *Shigella* in relation to other diseases and the structure of the health care system regarding early childcare.

The panel members were concerned about reducing vaccine schedule crowding and maximizing dosing schedule timing to ensure protection for children when they would be most vulnerable (at least up to 24 months, ideally up to 59 months). Many of the LMICs where children would potentially benefit most from a *Shigella* vaccine are already experiencing vaccine schedule crowding at standard Expanded Programme on Immunization (EPI) appointments. The panel members suggested three strategies to circumvent this concern. The first strategy was to vaccinate infants during a non-EPI well baby visit. While this approach may work for some countries, in others, often those with the highest burden, mothers usually cannot bring their infants for even one extra visit. Their infants already receive several vaccines at the visits they can make. The second suggested strategy was for the *Shigella* vaccine to be delivered in combination with another vaccine with a similar deployment schedule, such as the typhoid vaccine, which is given at six or nine months of age. Finally, a third potential strategy would be targeted vaccination of high-risk/high-burden areas. This prioritized deployment into populations with low WASH access and high *Shigella* burden may be the most beneficial strategy. In addition, as shigellosis is not limited to only low WASH access areas, the vaccine may also benefit and be used by other populations, such as travelers and military personnel and residents of other areas with high endemic shigellosis rates. Adoption of the vaccine by these other populations would ultimately defray its overall development cost, creating the possibility of tiered pricing and enabling lower prices for LMICs. However, as panel members asserted, it is still important to note that high-burden areas (e.g., Bangladesh) also have vaccine schedule crowding. These countries would still likely prefer a *Shigella* vaccine to be delivered with another vaccine or at least piggyback on an existing vaccination appointment.

**Session 1 synthesis**

The panel members agreed that the evidence supports a likely relationship between *Shigella*-attributable diarrhea and stunting (see Table 2 for consensus opinions and recommendations for both sessions). However, much of that relationship is complex and challenging to separate from other influential factors on child growth (e.g., adequate nutrition, access to health care and adequate WASH, prevalence of environmental enteropathy, and infections by other enteric pathogens).

**Table 2:** Discussion topics, consensus opinions, and recommendations by the expert panel.

**Discussion topics**	**Consensus opinion**	**Caveats**	**Recommendations**
**Session 1: The relationship between *Shigella* and childhood growth faltering and stunting**			
The relationship between *Shigella* infections and childhood growth faltering	A relationship between *Shigella* infections, even less severe ones, and linear growth faltering/childhood stunting exists.	Many of the causative mechanisms and related underlying processes of the *Shigella*-stunting relationship need better characterization. Few studies have shown the relationship between asymptomatic detections and growth faltering. No global estimates of asymptomatic *Shigella* infections exist.	While including less severe *Shigella*-attributable diarrhea in models assessing the growth impacts of children is acceptable, still inadvisable to include asymptomatic *Shigella* burden in impact or cost-effectiveness models.
Model inputs and assumptions for modeling the stunting impact of *Shigella* infections	While integrating new findings into *Shigella* models is desirable, well-established and replicated results are preferable to include in these models. Based on current evidence, it is doubtful that a *Shigella* vaccine would be able to provide sterilizing immunity.	No evidence to date that a *Shigella* vaccine could provide sterilizing immunity against *Shigella* infections. Studies on *Shigella* and linear growth use varying metrics and approaches to assign diarrheal etiology and symptomology and child linear growth outcomes, making it difficult to standardize the effect of *Shigella* infection across studies.	Modeling approaches that account for repeated infections and cumulative burden are preferred. Models should also account for infection duration, the impact of treating *Shigella* episodes with antibiotics on child growth, and co-infection with multiple enteric pathogens.
Vaccination timing and administration	Early *Shigella* vaccination would provide maximum protection from severe disease in infants and ideally prevent future stunting. Avoiding vaccine schedule crowding and timing doses to protect children during their most vulnerable period is essential for maximum protection of children from *Shigella*-associated short and long-term outcomes.	Many children from LMICs that would benefit the most from a *Shigella* vaccine already experience vaccine schedule crowding at Expanded Programme on Immunization appointments. While preventing *Shigella* infection through vaccination is expected to reduce childhood growth deficits, whether a vaccine can have such an impact is unknown.	Vaccinating infants at 6 and 9 months balances the benefits of protection with avoiding vaccine schedule crowding. Vaccinating children at 3 and 6 months may provide the best protection, especially in high *Shigella* burden settings. *Shigella* vaccination could be deployed at a non-EPI medical visit during infancy to avoid schedule crowding. Targeted or combination vaccine approaches can help protect those at highest risk while minimizing vaccine schedule crowding. Vaccine probe trials that include measures of growth faltering as *a priori* outcomes are needed.
**Session 2: Potential long-term economic benefits of preventing linear growth deficits by *Shigella* vaccination**			
Disentangling health and cognition in assessing economic impact of growth faltering	The effects of stunting from cognition cannot be currently disentangled.	No evidence to date that catch-up growth can entirely reverse or negate the effects of growth faltering during the first two years of life.	Height may be used as a partial proxy for cognition until their effects on economic earnings can be distinguished in economic models that include productivity. Catch-up growth does not need to be accounted for in an economic model of long-term stunting impacts.
Preferred growth faltering measure to use in impact and cost-effectiveness models	Using only stunting as a measure can underestimate the effects of linear growth faltering more broadly.	Linear growth faltering data do not cover enough countries to build large-scale models.	When possible, using linear growth faltering data is recommended. Limitations in global data sources may require using stunting as an outcome in global or large-scale models.
Long term vaccine impacts and introduction decisions	**Split consensus** Group 1: Health care decision-makers at governing bodies such as ministries of health are usually most interested in short-term health system costs and benefits related to vaccine introduction.Group 2: Government departments responsible for overall budget (e.g., Ministry of Finance) that allocate funds to ministries of health might be interested in broader economic benefits of health interventions.	More difficult to show empirical connection between vaccination and long-term health and non-health impacts. Ignoring long-term non-health gains can potentially underestimate the full value of health interventions,	*Shigella* vaccine impact and economic assessments should include two sets of outcomes—one using more traditional variables and another that includes the broader impacts beyond immediate health (e.g., future productivity). A multi-sectorial approach that involves a wider range of sectors in decision-making around health care investments with benefits falling outside of the health sector (for example, productivity gains from a vaccine) may draw resources from these different sectors.
Vaccine roll-out patterns	**Split consensus** Group 1 advocated for targeted vaccination in high-risk areas initially. Group 2 advocates for wide and immediate introduction of the vaccine.	Need to make sure burden of *Shigella* is sufficiently high to warrant a targeted approach. Vaccine effectiveness studies are needed that ideally show a reduction in childhood stunting upon *Shigella* vaccination.	Benefit-cost and cost-effectiveness analyses for interventions should assess at-risk (general population) and high-risk (prioritized groups) groups separately and compare the model results to guide vaccination decisions.

While existing data does provide some information about the long-term growth effects of *Shigella* infections and their downstream impacts, many of the causative mechanisms and related underlying processes need better characterization. Most panel members felt that the large epidemiological datasets and studies on this topic have already uncovered as much as possible. An ideal next step would be to conduct vaccine probe trials to address the remaining questions. Some panel members were concerned that while vaccines against a single enteric pathogen might result in minor improvements in linear growth effects among children in LMICs, it may inadvertently shift the attention away from WASH improvements. While WASH interventions are often expensive and time-consuming, they would likely reduce more pathogen-related linear growth deficits by limiting exposure to several pathogens. At the same time, other panelists suggested that further delays in developing this vaccine will result in preventable morbidity and mortality—taking timely action could save or improve many lives.

If a *Shigella* vaccine for young children was developed, the panel felt that vaccinating infants in their first year of life would maximize their protection from severe illness and ideally result in the most substantial reduction of stunting risk. While the panel offered plausible estimates for *Shigella* episode-associated decrements in LAZ score and an upper bound of the linear decrements associated with *Shigella* infection, the clinical significance of these estimates remains unknown. Future estimates may want to include the perspective of other relevant experts, such as nutritionists, who were not present at this meeting.

## Session 2. Potential long-term economic benefits of preventing linear growth deficits by *Shigella* vaccination

***Question 4. Suppose some economic gains associated with height are actually cognition-based rather than stature-based. Do you think that the proportion of economic gains due to each should be estimated individually? How can catch-up growth potentially impact this relationship?***

The panel largely agreed that based on current methods and data, it is currently not possible to disentangle the effects of stunting from cognition completely, and that height may be used as a partial proxy for cognition until their effects on economic earnings can be distinguished. Also, if cognition is the primary factor associated with wages in adulthood, and catch-up growth fails to bring catch-up cognition, then catch-up growth is irrelevant to long-term impacts. While children can and do experience catch-up growth, and some evidence links this catch-up growth with improved cognitive outcomes [34], most of it occurs after two years of age and cannot necessarily mitigate all of the consequences of early undernutrition. The panel decided this does not need to be accounted for in an economic model of long-term stunting impacts based on limited current knowledge.

**E** **mergent discussion theme: Preferred growth decrement outcomes in disease burden and vaccine impact analyses.**

Panel members discussed the advantages and drawbacks of different ways of representing child growth deficits in burden, impact, and economic models. Global health professionals generally use stunting in their models as a dichotomous measure. This approach’s advantage is that most existing data has been collected under these categories. However, nutrition and development experts usually focus on linear growth faltering in their studies, because the exclusive use of stunting can broadly underestimate the effects of linear growth faltering. Children also have adverse outcomes related to linear growth decrements that do not necessarily render them as having stunted growth. A recent review [38] by nutritionists advocates for discontinuing stunting as an outcome measure. However, while linear growth faltering data exist for several countries [44], they do not cover enough countries to build large-scale models, sometimes necessitating the use of childhood stunting as an outcome in global models.

***Question 5. Are the public health and economic benefits of Shigella vaccination discussed here likely enough to drive a country’s decision to vaccinate?***

Several panel members concurred that when ministries of health (or equivalent health care governing bodies) are considering new vaccine introductions, their primary concerns revolve around short-term health system costs and benefits, as their decision-making is often made within the context of the health care budget. For example, they are likely to focus on vaccine benefits in reducing hospitalizations, mortality, and direct medical costs rather than potential long-term benefits, especially non-health benefits, such as increased future productivity. Some members felt that reducing childhood stunting may be compelling enough for policymakers to consider introducing a *Shigella* vaccine. However, policymakers are still unlikely to make introduction decisions based on long-term productivity benefits, especially considering the limited empirical evidence of a causal link.

Other panel members suggested that in their experience, the government departments (e.g., ministries of finance) responsible for the overall budget and allocating funds to the ministries of health are often more interested in the broader benefits relative to the cost of a particular program or intervention. They pointed out that ministry of mealth budgets can be expanded by demonstrating the broader economic benefits of health interventions to ministries of finance. According to a previous analysis, 60% of the economic benefits accrued by reducing low birthweight were due to productivity gains in adulthood, compared to reduced health care costs or the value of averted mortality [45]. Therefore, ignoring any long-term non-health gains can potentially underestimate the full value of health interventions. Building on this idea, some panelists suggested a multi-sectorial approach that involves including a broader range of sectors in health care investment decision-making with benefits falling outside of the health sector (for example, productivity gains from a vaccine), ideally drawing resources from these different sectors. For example, ministries of education might be interested in investing in this intervention because it has implications for labor market outcomes. While this would be ideal for addressing this issue, especially in light of a recent push for co-financing among sectors receiving benefits from an intervention funded by the health sector [46], this approach is not very well-developed within countries. They often have a narrower focus on immediate health costs and benefits when making resource allocation decisions regarding the health sector.

Panelists advocated for two possible patterns of vaccine roll-out—initially targeting vaccination in high-risk areas before broader roll-out or introducing the vaccine immediately. Based on their country-specific experiences, some panel members highlighted the need for a targeted approach aimed at most at-risk populations. This option would be particularly appealing for certain South Asian countries (e.g., India, Bangladesh, and Nepal) for any additional enteric childhood vaccines. However, before advocating for targeted *Shigella* vaccination, two requirements were suggested: (1) an assessment of the *Shigella* burden areas with high-risk populations to ensure that the burden is sufficiently high to warrant a targeted approach and (2) conducting effectiveness studies that ideally show a reduction in childhood stunting upon *Shigella* vaccination. Another panel member suggested that as interventions improve and the incidence of infectious diseases decreases in overall populations, as has been the case in the past 30 years, preventative interventions such as vaccination will likely be prioritized for populations from high-risk regions and areas [47]. Therefore, assessing the benefit-cost and cost-effectiveness of interventions for at-risk (general population) and high-risk (prioritized groups) populations will likely be a critical approach for future economic analysis of vaccine-based interventions.

Panel members who supported wider *Shigella* vaccine deployment pointed out that poverty, while a dominant risk factor, was not the only one for shigellosis. Higher SES areas still may have endemic shigellosis because of transmission by flies or other environmental risk factors and may benefit from the vaccine. Also, some regions of the world with better access to WASH and lower enteric burden than other LMICs (e.g., Latin America) may consider introducing a *Shigella* vaccine because their population still experiences gastroenteritis and shigellosis. Providing broader distribution and availability of the vaccine could lead to higher demand and production, subsequent reduction of vaccine cost, and ultimately make it easier for countries eligible for support from Gavi, the Vaccine Alliance, and others to use the vaccine.

**Session 2 synthesis**

The panel largely agreed that height can function as a partial proxy for cognition in economic models that included productivity until their effects on economic earnings can be distinguished. While using linear growth instead of stunting in *Shigella* burden, impact, and productivity models may encompass a more extensive range of *Shigella*’s effects on child growth, some models (e.g., global models) may need to rely on stunting as an outcome measure because of data limitations. *Shigella* vaccine impact and economic assessments should include two sets of outcomes—one using more traditional variables and another including the broader impacts beyond immediate health (e.g., future productivity). This approach will ensure that models are accessible and reflect the interests of health care decision-makers while also providing them with a broader picture of the potential impacts of *Shigella* vaccination. Several suggestions were made for further nuancing vaccine impact and economic models, including adjusting for regional unemployment rates, using dynamic disease transmission models to simulate direct and indirect health benefits, and modeling noncommunicable diseases as part of a sensitivity analysis (although data are limited). While some panel members felt that certain countries would only be interested in deploying the vaccine in high-risk areas, others suggested that widespread roll-out would reduce costs overall and make it available for populations of higher-income regions with endemic *Shigella*.

## Knowledge gaps and future directions

Of the many significant knowledge gaps that exist—despite the extensive investments in diarrheal disease research during the past two decades, which has dramatically enhanced our understanding of diarrheal incidence, etiology, and adverse consequences—the panel felt that the remaining gaps could be best addressed by data collected post-vaccine introduction or through large clinical trials. The most critical question that a vaccine trial may answer is whether preventing *Shigella* infection ameliorates childhood stunting or growth faltering. If it can, then the long-term impacts of these vaccines can be explored in depth. However, these trials should be run in high-burden settings to ensure they capture the potential growth effects that might not be achievable in other populations. Also, assessing whether the currently used predictors of *Shigella* correctly forecast areas with a high *Shigella* incidence would be crucial to ensure that the right populations are receiving the vaccine.

While studies show some evidence linking enteric infections [48], [49], [50] and childhood stunting [10], [11], [12], [51] to future increased noncommunicable diseases (NCDs; e.g., obesity, body mass index) and reduced productivity, this relationship needs to be better characterized for *Shigella* infections. These studies support a qualitative relationship between diarrheal diseases and NCD risk; however, there may not be enough empirical evidence to calculate accurate global population estimates of a *Shigella* vaccine-attributable reduction of adult NCD burden.

As mentioned previously, *Shigella* is not the only pathogen linked to linear growth faltering. However, a vaccine probe or similar study is needed to determine the magnitude of the impact of removing one stunting-related pathogen on childhood growth faltering. Also, recent studies found that intestinal microbiome perturbations, in which excessive levels of oropharyngeal bacteria are located in the small intestine (known as small intestinal bacterial overgrowth or SIBO), are linked to childhood stunting [52], [53]. The connection between gut microbiota, enteric infections, and linear growth faltering is complex. Ideally, eliminating some of these pathogens and potentially SIBO and closely monitoring social and environmental factors may help determine the best way to prevent these undesirable and lingering outcomes.

Finally, as some countries may prefer a targeted vaccine deployment approach, future cost-effectiveness models could also model the cost, cost-effectiveness, and cost-benefit of vaccinating at-risk populations versus high-risk populations. Exploring the cost of vaccinating all versus vaccinating the most vulnerable may help clarify which approach will most likely be adopted while accounting for protecting those who need it most. As part of this investigation, researchers should also examine the implications for countries that would take an at-risk introduction strategy versus a high-risk introduction strategy.

## Declaration of Competing Interests

Dr. Kotloff receives funding from Institut Pasteur to conduct Shigella vaccine clinical trials. Dr. Lanata is a member of the World Health Organization (WHO) COVID-19 vaccine effectiveness working group and WHO Product Development Advisory Group. All other authors have no competing interests to declare.
